# Analysis of the N-terminal region of human MLKL, as well as two distinct MLKL isoforms, reveals new insights into necroptotic cell death

**DOI:** 10.1042/BSR20150246

**Published:** 2016-01-22

**Authors:** Katja Hrovat Arnež, Michaela Kindlova, Nilesh J. Bokil, James M. Murphy, Matthew J. Sweet, Gregor Gunčar

**Affiliations:** * Department of Chemistry and Biochemistry, Faculty of Chemistry and Chemical Technology, University of Ljubljana, SI-1000, Ljubljana, Slovenia; † Institute for Molecular Bioscience, IMB Centre for Inflammation and Disease Research, and Australian Infectious Diseases Research Centre, The University of Queensland, Brisbane, Queensland 4072, Australia; ‡ Cell Signalling and Cell Death Division, The Walter and Eliza Hall Institute of Medical Research, Parkville, Victoria 3052, Australia; § Department of Medical Biology, University of Melbourne, Parkville, Victoria 3050, Australia

**Keywords:** cell death, isoform, macrophage, mixed lineage kinase domain-like, MLKL, necroptosis

## Abstract

The pseudokinase mixed lineage kinase domain-like (MLKL) is an essential effector of necroptotic cell death. Two distinct human MLKL isoforms have previously been reported, but their capacities to trigger cell death have not been compared directly. Herein, we examine these two MLKL isoforms, and further probe the features of the human MLKL N-terminal domain that are required for cell death. Expression in HEK293T cells of the N-terminal 201 amino acids (aa) of human MLKL is sufficient to cause cell death, whereas expression of the first 154 aa is not. Given that aa 1–125 are able to initiate necroptosis, our findings indicate that the helix that follows this region restrains necroptotic activity, which is again restored in longer constructs. Furthermore, MLKL isoform 2 (MLKL2), which lacks much of the regulatory pseudokinase domain, is a much more potent inducer of cell death than MLKL isoform 1 (MLKL1) in ectopic expression studies in HEK293T cells. Modelling predicts that a C-terminal helix constrains the activity of MLKL1, but not MLKL2. Although both isoforms are expressed by human monocyte-derived macrophages at the mRNA level, MLKL2 is expressed at much lower levels. We propose that it may have a regulatory role in controlling macrophage survival, either in the steady state or in response to specific stimuli.

## Abstract (precis)

We show that mixed lineage kinase domain-like (MLKL) isoform 2, which lacks the pseudokinase domain and activation loop phosphorylation sites, is a more potent activator of cell death compared with MLKL isoform 1. Both MLKL isoforms are expressed in human monocyte-derived macrophages.

## INTRODUCTION

Necroptosis is a form of programmed necrotic cell death that is triggered by specific inflammatory stimuli such as tumour necrosis factor (TNF) [1] and Toll-like receptor (TLR) agonists [2] when caspase-8 activity is concomitantly inhibited. The lytic nature of necroptosis results in damage-associated molecular patterns (DAMPs) release [3], thus triggering potent innate immune cell activation and inflammation. Consequently, necroptosis has been linked to inflammation-linked pathology in the intestinal tract [4–6], skin [5,7], eye [8], cardiovascular system and brain [9]. Although specific contributions of necroptosis to human diseases are still emerging, there has been great interest in molecular mechanisms initiating this process.

Initiation of necroptosis depends on the tightly regulated interactions between two serine threonine kinases, receptor interacting protein kinase 1 (RIP1) and RIP3, their kinase activity and their phosphorylation [1,10–12]. The complexity of this pathway was highlighted by a recent study showing that RIP1 can inhibit necroptosis during developmental processes and that RIP3-dependent necroptosis can be activated independently of RIP1 [5]. The mixed lineage kinase domain-like (MLKL) pseudokinase is a RIP3 substrate and an essential molecular component of necroptosis [13,14]. RIP3 phosphorylates MLKL at Thr-357 and Ser-358 in human [13], and at Ser-345, Ser-347 and Thr-349 in mouse [15]. These residues are present within the activation loop [16] in the C-terminal pseudokinase domain of MLKL. Phosphorylation is believed to induce a conformational change, releasing the N-terminal four-helix bundle (4HB) domain [16], which is indispensible for necroptotic activity [16–18]. Once activated, MLKL can form oligomers that translocate to the plasma membrane [19]. It has been proposed to disrupt membrane integrity by increasing calcium [20] or sodium influx [18], by compromising membranes or forming transmembrane pores [17,21,22], but the exact mechanism(s) by which MLKL elicits cell death remain elusive.

Human HT-29 cells were reported to express two distinct MLKL isoforms, which are generated by alternative splicing [14]. Isoform 1 (MLKL1) consists of an N-terminal 4HB domain and a C-terminal pseudokinase domain, which are connected by a two-helical brace region [15,23]. Isoform 2 (MLKL2) has the same N-terminus as MLKL1, but lacks the universal kinase core domain, including two RIP3 phosphorylation sites on the activation loop. Since the first 125 N-terminal amino acid (aa) residues of MLKL, which encode the 4HB domain, have been shown to trigger cell death [16,17], our aim was to explore the ability of different MLKL constructs to induce cell death, including MLKL2, which may have distinct biological functions to MLKL1.

## MATERIALS AND METHODS

### Constructs

Plasmids encoding human MLKL1 and MLKL2 were purchased from DNASU Plasmid Repository and Geneservice, respectively. Coding regions for MLKL1, N-201 (aa 1–201) and C-terminal construct MLKL C-terminal domain (C1) (aa 155-471) were cloned into pcDNA3.1 vector (Life Technologies) using KpnI and XhoI (NEB) restriction sites. The construct encoding MLKL2 was used for cloning of the N-154 (aa 1–154) and N-166 (aa 1–166) constructs and for full-length MLKL2 into the pcDNA3.1 plasmid, using BamHI and XhoI (NEB) restriction sites. All constructs were sequence verified. Plasmid DNA was purified using Endofree Maxi kits (Qiagen) for use in cell-based assays. Primer sequences used for cloning are available upon request.

### Cell culture

HEK293T cells, obtained from the A.T.C.C., were cultured in DMEM medium (Life Technologies) supplemented with 10% FBS (Life Technologies), 1% GlutaMAX-I (Life Technologies), penicillin (50 units/ml) and streptomycin (50 μg/ml) (Life Technologies) in a humidified atmosphere of 5% CO_2_ and 37°C. Human CD14^+^ monocytes were isolated from buffy coats obtained from the Australian Red Cross Blood Service using a MACS CD14^+^ positive selection kit (Miltenyi Biotech), according to the manufacturer's protocol. Cells were then differentiated into human monocyte-derived macrophages (HMDM) using either 1×10^4^ U/ml CSF-1 (Chirion) or 10 ng/ml GM-CSF (PeproTech) for 6 days. On day 6, HMDM were harvested and plated out at the appropriate density in RPMI 1640 medium (Life Technologies) supplemented with 10% FBS (Life Technologies), 1% GlutaMAX-I (Life Technologies), penicillin (50 units/ml) and streptomycin (50 μg/ml) (Life Technologies) in the presence of growth factor. Approval for all experiments using primary human cells was obtained from the University of Queensland Medical Research Ethics Committee.

### Transient transfection of HEK293T

One day prior to transfection, HEK293T cells were seeded at 2×10^5^ cells per well in 12-well plates for LDH assays, or at 1×10^6^ cells per well in six-well plates for generating cell lysates for immunoblotting. For transient transfection experiments, Lipofectamine 2000 (Life Technologies) was used, according to the manufacturer's protocol.

### Lactate dehydrogenase (LDH) release assay

The effects of MLKL overexpression on cell viability of HEK293T cells was determined by lactate dehydrogenase release assays, as previously described [24]. Supernatants of transfected cells were collected at 24 h post-transfection, centrifuged for 5 min at 500 ***g*** and LDH release was measured using the LDH Cytotoxicity Assay kit (Sigma–Aldrich), according to the manufacturer's protocol. Total cellular LDH was determined by lysis of HEK293T cells with 0.1% Triton X-100. The absorbance at 490 nm was measured using a Powerwave XS microplate reader (Bio-TEK), and results are presented as the percentage of the total LDH released from cells.

### Western blot analysis

Whole cell extracts were lysed on ice in RIPA buffer (150 mM NaCl, 50 mM Tris/HCl pH 7.4, 1% NP-40, 0.1% SDS), supplemented with complete EDTA-free protease inhibitor cocktail (Roche). Total protein concentrations were quantified using the BCA protein kit (Life Technologies), and cell lysates containing 20 μg of protein were subjected to electrophoretic separation on denaturing polyacrylamide gels under reducing conditions, followed by transfer to PVDF membranes. The latter were then probed with a mouse anti-myc antibody (1:1000, Cell Signalling Technologies), a rat anti-MLKL antibody (1:2000) [15] and a rabbit anti-GAPDH antibody (1:2500, Trevigen), followed by the appropriate secondary horseradish peroxidase-conjugated antibody (1:3000, Cell Signalling Technologies). The signal was visualized using the chemiluminescent ECL reagent (Life Technologies).

### RNA preparation and quantitative PCR analysis of gene expression

CSF-1 or GM-CSF-differentiated HMDM were seeded on to six-well plates at a density of 1×10^6^ cells per well. Total RNA was purified using a Research RNA purification mini kits (Zymo Research) and treated with DNase I (Life Technologies). Superscript III reverse transcriptase (Life Technologies) and oligo-dT primers were used to reverse transcribe RNA into cDNA. Quantitative RT-PCR was performed using SYBR Green (Life Technologies) with the Viia7 (Life Technologies) detection system. All samples were analysed in technical triplicate and results were expressed relative to the reference gene *HPRT*. Primer sequences used for detection of *MLKL1*, *MLKL2* and *HPRT* are available upon request. The specificity of *MLKL1* and *MLKL2* qPCR primers was confirmed by melt curve analysis and by verifying the size of qPCR amplicons using agarose gel electrophoresis.

### Molecular modelling

The model of full-length MLKL1 was generated using Modeller [25] with the structures PDB: 2MSV [22], PDB: 4MWI [23] and PDB: 4BTF [15] as templates. The MLKL2 model was generated using the I-TASSER modelling server [26]. All images were prepared using PyMOL (DeLano Scientific LLC).

### Phosphorylation site prediction

Phosphorylation sites were predicted using the online prediction servers NetPhos 2.0 [27] and Predikin [28]. Predictions with Predikin were made for both MLKL isoforms as RIP3 kinase substrates.

### Statistical methods

Combined data from multiple independent experiments and containing at least three variables were subjected to ANOVA analysis, followed by Dunnett's Multiple Comparison test. A two-tailed unpaired *t* test was used for comparing two data points. *P*-values of <0.05 were considered as statistically significant.

## RESULTS

### MLKL2 has enhanced cell killing activity relative to MLKL1

**Figure 1:**
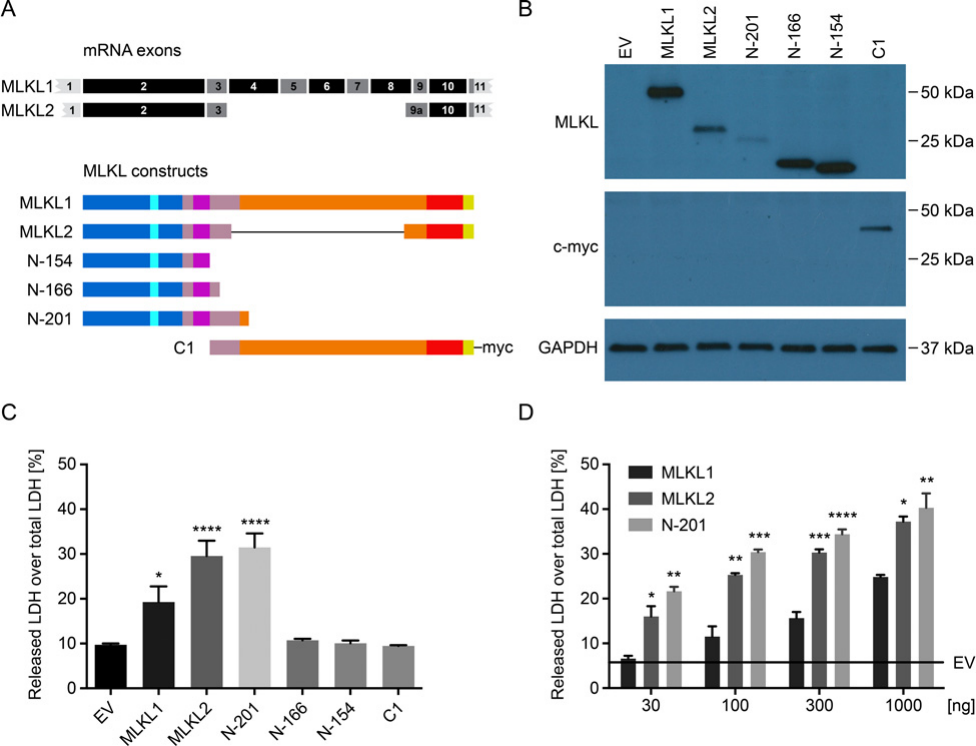
Human MLKL2 is a more potent inducer of cell death than MLKL1, and the N-154 construct has no biological activity when expressed ectopically (**A**) Schematic representation of the *MLKL1* and *MLKL2* mRNA transcripts, and different human MLKL expression constructs used for overexpression in HEK293T cells. Colour codes are as in Figure 2. (**B**) HEK293T cells were transfected with different human MLKL constructs (100 ng) or an empty vector (EV) control (100 ng). Total cell lysates were collected at 12 h post-transfection and subjected to immunoblotting. GADPH was used as a loading control. Data are representative of three independent experiments. (**C**) HEK293T cells were transfected with EV or human MLKL expression constructs, as shown in (**A**). Cell viability was determined at 24 h post-transfection by LDH release assays. Shown is the mean+S.E.M. of six independent experiments. (**D**) HEK293T were transfected with increasing amounts of expression plasmids encoding human MLKL1, MLKL2 or N-201, with the total amount of transfected DNA being kept constant with EV. LDH release was measured at 24 h post-transfection. Shown is mean+S.E.M. of three independent experiments. *P≤0.05, **P≤0.01, ***P≤0.001, ****P≤0.0001.

**Figure 2:**
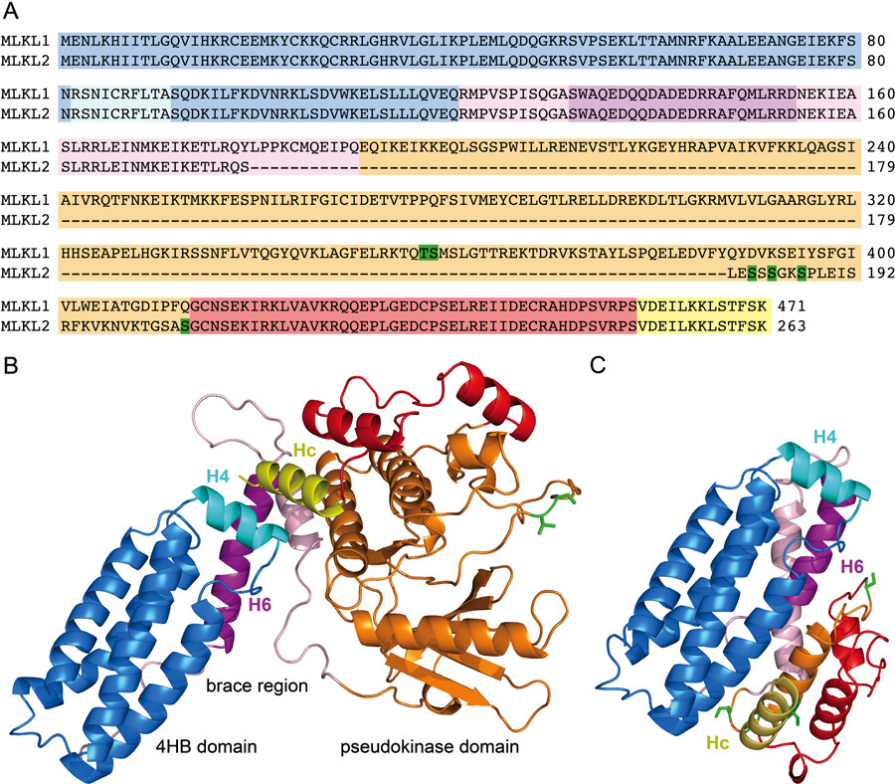
Human MLKL2 lacks the major part of the kinase-like domain that is present in MLKL1 The N-terminal 4HB is shown in blue, the brace region in pink and the pseudokinase domain in orange, red and yellow. The regions that differ in MLKL1 compared with MLKL2 are shown in orange. Known phosphorylation sites in MLKL1 and putative phosphorylation sites in MLKL2 are shown in green. Helices H4, H6 and MLKL C-terminal helix (Hc) are shown in cyan, magenta and yellow, respectively. (**A**) Sequence comparison of MLKL1 and MLKL2. (**B**) Human MLKL1 model. (**C**) Human MLKL2 model.

Two alternatively spliced isoforms of MLKL were reportedly expressed by HT-29 colon human adenocarcinoma cells [14]. *MLKL1* encodes the full-length 471 aa long MLKL isoform, whereas *MLKL2* lacks exons 4–8, has a longer version of exon 9, and encodes a shorter 263 aa long protein lacking much of the pseudokinase domain (Figure 1A). To determine whether there are differences in their biological activities, we ectopically expressed the two isoforms, as well as a number of N-terminal constructs of different length and a C-terminal construct (Figure 1A), in HEK293T cells. All proteins, except the C-terminal construct were expressed without any epitope tags. Since the monoclonal anti-MLKL antibody used in the study by Murphy et al. [15] recognizes the brace region of MLKL (see Figure 2B), a c-myc tag was used for detection of the expressed C-terminal construct. The MLKL1, N-166 and N-154 constructs were found to be expressed at similar levels, whereas MLKL2 and N-201 were expressed at much lower levels in HEK293T cells (Figure 1B). To investigate whether this correlated with MLKL2 and N-201 triggering more pronounced cell death, the effects of expression of the different constructs on LDH release were next assessed. Indeed, the longest N-terminal MLKL construct tested (N-201) initiated cell death when overexpressed in HEK293T cells, whereas the shorter N-terminal constructs and the C-terminal construct did not. Moreover, MLKL1 was much less effective than MLKL2 in triggering cell death (Figure 1C). These conclusions are supported by analysis over a concentration range, where MLKL2 was always more active than MLKL1 (Figure 1D).

### Models of human MLKL1 and MLKL2

To gain insights into potential mechanisms that might account for differences in the activities of MLKL1 and MLKL2, models of both isoforms were generated (Figures 2A–2C). The crystal structure of murine MLKL revealed a 4HB domain attached to the typical kinase-like fold with a two helix linker [15]. Since both the N-terminal domain NMR structure [22] and the C-terminal domain X-ray structure of human MLKL isoform 1 [23,29] were determined separately, we used the structure of the full-length murine protein to position them accordingly. The NMR structure of the human N-terminal domain has revealed that there is one additional helix (H4) that is located on the top of the 4HB, which was not visible in the electron density of the full-length murine structure [22]. Our model of human MLKL1 reveals that helix H4 interacts with, and fits between, helix H6 and the very last helix of the C-terminal pseudokinase domain (Figure 2B). The model of MLKL2, in contrast, is missing the core kinase-like domain, whereas three α-helices, which are not part of the universal kinase core, are present in the C-lobe of the C-terminal domain. The I-TASSER server predicts that these three α-helices are adjacent to the N-terminal 4HB and brace regions, making MLKL2 much more compact than MLKL1. In this model, and in contrast with the human MLKL1 model, the terminal C-lobe helix no longer interacts with helix H4 (Figure 2C).

### Prediction of phosphorylation sites

We analysed possible phosphorylation sites on residues of the shorter MLKL2 that are not present in MLKL1 as a consequence of alternative splicing and a different reading frame in exon 9. Within this short region of 27 aa, there are seven serine residues and one threonine residue. The NetPhos 2.0 server identified serine residues 182, 184, 187 and 205 as the most likely candidates for phosphorylation. Of these, Ser-182, with a relative score of 68%, was identified by Predikin server as the most likely candidate for phosphorylation by RIP3. For comparison, the prediction scores for the experimentally determined Ser-358 and Thr-357 phosphorylation sites in MLKL1 [13] are 75% and 67%, respectively.

### MLKL1 is expressed at higher levels as compared with MLKL2 in HMDM

**Figure 3:**
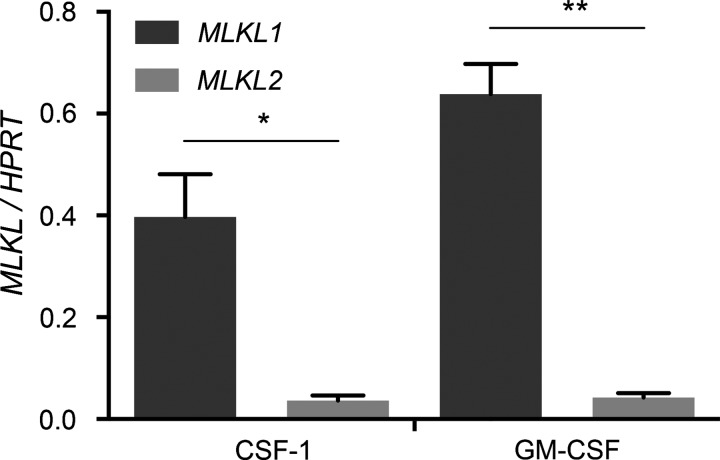
MLKL1 is expressed at higher levels than MLKL2 in HMDM Quantitative PCR primers specifically detecting human *MLKL1* and *MLKL2* were used to quantify mRNA levels relative to *HPRT* in CSF-1- and GM-CSF-derived HMDM. Data represent mean+S.E.M. of three independent experiments *P≤0.05, **P≤0.01; by two-tailed unpaired *t* test.

The observed difference in cell killing activity between the two MLKL isoforms led us to investigate their expression patterns. Since macrophages are exquisitely sensitive to TLR ligands and TNF, both of which can trigger necroptosis upon loss of caspase-8 function, we assessed expression of each isoform in HMDM. Using quantitative real-time PCR with transcript-specific primers, we found that *MLKL1* and *MLKL2* are expressed by both CSF-1- and GM-CSF-derived HMDM at the mRNA level. However, basal levels of *MLKL1* mRNA were ∼10-fold higher than those of *MLKL2* (Figure 3).

## DISCUSSION

Although MLKL is essential for necroptosis [13,15,30], the exact mechanisms by which it initiates this process remain unclear. The N-terminal 4HB domain of both human and mouse MLKL (aa 1–125) is sufficient to trigger necroptosis [16–18], and the same has been demonstrated for N-terminal regions spanning the first 180 or 210 aa of human MLKL [17], and 180 aa for mouse MLKL [16]. Somewhat surprisingly, the expression of the N-terminal 167 residues was reported to have no necroptotic activity [17]. Similarly, we found that N-166 did not trigger cell death, whereas the N-201 construct was active (Figure 1C). Su et al. [22] presented the NMR structure of the first 2–154 aa of human MLKL, and reported that the encoded protein induced liposome leakage, although more slowly than the 4HB construct, leading to the conclusion that H6 α-helix, the first helix of the ‘brace’ region, inhibits this activity. In our experiments, we observed that neither the N-154 nor N-166 construct triggered cell death in HEK293T cells. Since both constructs include the H6 α-helix, our findings support the view that this region constrains necroptotic activity. Necroptotic activity is restored in longer constructs (180, 201, 210 aa), suggesting that a region C-terminal to the helix H6 is needed to facilitate pulling this helix away from the 4HB, thus unleashing its activity. Similarly, activation of mouse MLKL occurs by phosphorylation of the pseudokinase domain activation loop, which liberates 4HB, causing cell death [16].

In human MLKL, residues Thr-357 and Ser-358 are phosphorylated [13,21], and their mutation inhibits the necroptotic activity of MLKL1 [13]. The alternatively spliced MLKL2 is 208 aa shorter than MLKL1, and lacks a substantial part of the pseudokinase domain, including the identified phosphorylation sites [13,14]. Hildebrand et al. [16] proposed that the C-terminal pseudokinase domain acts as a suppressor of MLKL-mediated cell death until activated. This probably explains the enhanced capacity of MLKL2, as compared with MLKL1, to initiate cell death. Our molecular models provide a plausible explanation for these differences. In the MLKL1 model (Figure 2B), the core C-lobe of the kinase-like domain structurally supports the last C-terminal helix that stabilizes the helices H4 and H6. This is not the case in the MLKL2 model (Figure 2C) and, even if the positioning of the C-terminal helix is not predicted correctly, it is unlikely that it would adopt the same position as in MLKL1, without the aid of the missing core kinase-like domain. Helix H4 is evidently important for the effector function of MLKL, since binding of necrosulfonamide to Cys86 in the middle of helix H4 inhibits necroptosis [13], as well as the liposome permeabilization activities of MLKL1 [21] and MLKL (2–154) [22]. The hydrophobic face of helix H4 is stabilized by helix H6, which as discussed above, inhibits this activity. The lack of interactions between the last C-terminal helix with helices H4 and H6 in MLKL2 probably explains the more potent biological activity of MLKL2. It also suggests that destabilization of this region is important for triggering MLKL effector function. Thus, the absence of the core kinase-like domain in MLKL2 may render it necroptotically active without requiring RIP3-dependent activation, as was observed when the 4HB domain alone was expressed in cells [16,17]. Alternatively, the activation of MLKL2 may depend on some yet to be identified phosphorylation event. A recent study has identified additional residues outside the activation loop in mouse MLKL that are subject to phosphorylation, which can enhance or even suppress MLKL activation [31]. There are eight possible phosphorylation sites, in the short 27 aa region that is unique to MLKL2, any of which might contribute to their fine tune MLKL2 necroptotic activity. It thus remains of interest to determine whether phosphorylation of one or more of these residues can modulate MLKL2-induced cell death and, if so, which kinases are responsible for these events.

In the presence of pan-caspase inhibitors, some TLR agonists [30], as well as bacterial pathogens [32], trigger necroptosis of mouse macrophages. Necroptosis has not been well studied in human macrophages, although a single publication showed that treatment with LPS and caspase inhibitors did cause cell death [33]. We find that both MLKL isoforms are expressed in human macrophages and that the basal expression of MLKL1 mRNA is ∼10-fold higher than that of MLKL2. Whether this level of MLKL2 expression is sufficient to regulate survival of macrophages in the steady state is unknown. It may be that other stimuli up-regulate MLKL2 expression and/or promote MLKL2 serine phosphorylation in macrophages to unleash its biological activity. Such a conclusion is supported by the findings of a previous study; in a comparison of HT-29 clones with either MLKL1 or both MLKL1 and MLKL2 stably silenced by shRNAs, knockdown of MLKL1 alone was sufficient to protect against TNF-induced necroptotic cell death [14]. This suggests that MLKL1 has a dominant role in TNF-triggered necroptosis, at least under these experimental conditions. Future studies should therefore address the external stimuli and intracellular signalling events that promote MLKL2 expression and/or activation.

## Abbreviations

aa: amino acid
EV: empty vector
4HB: four-helix bundle
HMDM: human monocyte-derived macrophages
MLKL: mixed lineage kinase domain-like
MLKL1: MLKL isoform 1
MLKL2: MLKL isoform 2
N-154: MLKL N-terminal domain of 154 amino acid residues
N-166: MLKL N-terminal domain of 166 amino acid residues
N-201: MLKL N-terminal domain of 201 amino acid residues
RIP: receptor interacting protein kinase
TNF: tumour necrosis factor
TLR: Toll-like receptor
